# Oral health-related quality of life in head and neck cancer survivors in Egypt within the first year after radiotherapy: a multivariable analysis

**DOI:** 10.1186/s12903-025-07488-9

**Published:** 2025-12-27

**Authors:** Fatma. E.A. Hassanein, Asmaa Abou-Bakr, Hany William, Yousra Ahmed, Suzan S. Ibrahim

**Affiliations:** 1 Oral Medicine, Periodontology, and Oral Diagnosis, Faculty of Dentistry, King Salman International University, El-tor, Egypt; 2 Oral Medicine and Periodontology, Faculty of Dentistry, Galala University, Suez, Egypt; 3 Department of oncolog, Ahmed Maher Teaching Hospital, Cairo, Egypt; 4 Prosthodontics Dentistry, Faculty of Dentistry, King Salman International University, El Tur, South Sinai Egypt; 5 Oral Medicine and Periodontology, Faculty of Dentistry, Ain Shams University, Cairo, Egypt; 6 Faculty of Oral and Dental Medicine, Nahda University in Egypt, Beni- Suef, Egypt

**Keywords:** Head and neck cancer, Oral health, Quality of life, Radiotherapy, EORTC QLQ-H&N35, Egypt

## Abstract

**Background:**

Radiotherapy for head and neck cancer (HNC) is frequently associated with debilitating oral complications that severely impact oral health-related quality of life (OHRQoL) in cancer survivors. Although survivorship care is gaining more attention worldwide, there remains a scarcity of data on OHRQoL in low-resource countries. Therefore, we aimed to evaluate OHRQoL among Egyptian head-and-neck cancer survivors during the first year following treatment and identify sociodemographic and clinical predictors influencing symptom severity using the EORTC QLQ-H&N35 tool.

**Methods:**

This cross-sectional study included 180 HNC survivors within 12 months post-treatment at Ahmed Maher Teaching Hospital, Cairo, Egypt. Participants completed the Arabic EORTC QLQ-H&N35 questionnaire, alongside structured interviews and clinical oral examinations (OHI-S and oral dryness scoring). Data were analyzed using descriptive statistics and multivariate regression (GLM-MANOVA) to determine predictors of OHRQoL impairment.

**Results:**

The most severe patient-reported symptoms were dry mouth (mean 53.7 ± 16.6), limited mouth opening (45.4 ± 22.2), and dental-related problems (37.9 ± 18.6). Multivariable analysis identified tumor site and financial status as independent predictors of greater symptom burden, while tooth-brushing frequency showed a marginal association (*p* = 0.05). Patients with oral cavity tumors and those with lower socioeconomic status consistently reported poorer OHRQoL across multiple symptom domains.

**Conclusions:**

This is the first study in Egypt using the EORTC QLQ-H&N35 tool to evaluate OHRQoL in HNC survivors in a comprehensive manner. One year after treatment, Egyptian HNC cancer survivors experience significant OHRQoL impairments, particularly associated with dry mouth, limited mouth opening, and dental pain. Tumor location, socioeconomic factors, and oral hygiene practices significantly influence symptom severity, highlighting the need for targeted supportive care interventions, including structured dental support and rehabilitation strategies tailored for at-risk patient populations in low-resource settings.

**Trial registration:**

The study was registered retrospectively on ClinicalTrials.gov (NCT06901401) on March 23, 2025.

## Introduction

Over 660,000 new cases and 325,000 fatalities from head and neck cancer (HNC) occur each year, making it the seventh most frequent malignancy worldwide [1–3], and its incidence is projected to rise by about 62% by 2035 [4]. In Egypt, HNC is a serious public health concern, accounting for 17% and 20% of all cancer cases [5, 6].

Radiation therapy (RT), a cornerstone of HNC management, continues to inflict acute and chronic toxicities that can be as debilitating as the disease itself [7, 8]. Local epidemiological data from particular Egyptian institutions show that up to 93% of HNC cases in some university hospitals require RT [9, 10].

Cancers and their treatment frequently compromise structures essential for speaking, chewing, and swallowing [11, 12], and RT-related oral mucositis, xerostomia, dysphagia, and taste loss often precipitate malnutrition, psychological distress, and overall quality-of-life deterioration [13].

Since oral health is essential to systemic well-being [14–16]. Disruptions in key oral functions pose a disproportionate threat to survivors’ daily functioning and social integration. Oral-health-related quality of life (OHRQoL) is therefore defined as the absence of any oral-condition–induced limitation on functional, social, or psychological activities [14, 17]. Patients with oral tumors routinely report some of the worst OHRQoL scores across all HNC subsites [18], making systematic QoL assessment indispensable for monitoring disease outcomes and shaping rehabilitative services [19]. The European Organization for Research and Treatment of Cancer Quality of Life Questionnaire Head and Neck 35 (EORTC QLQ-H&N35) is the most widely used, validated instrument for this purpose. It was selected for its superior specificity in capturing the detailed, treatment-related symptom burden of HNC patients compared to other instruments (such as FACT-H&N and OHIP-14) [20], as well as its reliability in long-term and cross-cultural settings, highlighting its value in personalized, multidisciplinary care planning [21].

Data on the oral side effects of RT in HNC survivors remains scarce in low- and middle-income regions. In Egypt, organized dental support before, during, and after RT is still largely absent, with limited awareness of RT-related oral toxicities means that only a minority of patients undergo systematic dental assessment, receive regular hygiene counseling, or access rehabilitative follow-up [22]. As a result, oral complications often linger well beyond the end of cancer therapy, eroding survivors’ OHRQoL and, ultimately, their overall OHRQoL [23, 24].

Interestingly, no previous study in Egypt has evaluated the oral symptom burden in this population using the validated EORTC QLQ-H&N35 tool. This study, therefore, aims to fill a significant gap by examining how these scores relate to sociodemographic factors, oral hygiene practices, and clinical parameters. We hypothesized that poorer clinical oral status and less frequent hygiene practices would be correlated with lower OHRQoL scores.

## Subjects and methods

### Study design

A cross-sectional, descriptive study was conducted and reported according to the STROBE guidelines (Strengthening the Reporting of Observational Studies in Epidemiology).

### Setting

The study took place at the outpatient oncology clinics of Ahmed Maher Teaching Hospital, Cairo, Egypt. Data collection occurred from October 2024 through April 2025. Clinical oral examinations and interviews were conducted in a designated dental operatory adjacent to the oncology follow-up clinics.

### Sample size calculation

The sample size for this study was determined using a two-sided one-sample t-test to estimate the number of participants required to detect a clinically meaningful difference in mean EORTC QLQ-H&N35 scores. Based on prior findings from Qamar et al. (2024) [24], who reported a mean score of 25.02 with a standard deviation of 15.86, we assumed a minimal clinically important difference of 4 units. At a 5% significance level (α = 0.05) and 80% power (1 − β = 0.80), the minimum required sample size was calculated as 124 participants [25]. To account for a 20% potential dropout, the final recruitment target was increased to 155 participants, ensuring adequate statistical power and reliability. Sample size calculation was performed using the Statsmodels library in Python (version 3.10) for Windows .

### Ethical considerations

The study protocol (HAM00213) was approved by the Ahmed Maher Medical Research Ethics Committee. All procedures conformed to the Declaration of Helsinki, and participants gave written informed consent before enrolment. Data were stored on encrypted, password-protected servers accessible only to the research team.

### Participants

Adult head-and-neck cancer (HNC) survivors were consecutively screened during routine post-treatment follow-up visits, using the oncology clinic appointment lists [26]. The survivorship clinic operates three days per week, and all eligible survivors attending during the study period were approached for participation.

#### Eligibility criteria were

(i) age ≥ 18 years, (ii) completion of cancer treatment (surgery, radiotherapy, chemotherapy, or combination) within the preceding 12 months, (iii) retention of at least one incisor and one molar in each dental arch (required for Oral Hygiene Index–Simplified [OHI-S]), and (iv) inter-incisal mouth-opening ≥ 20 mm.

#### Exclusion criteria

Included cognitive impairment preventing consent, severe systemic illnesses contraindicating oral examination, trismus < 20 mm, and consistent use of prescribed supportive drugs for oral sequelae (such as systemic analgesics for oral pain, saliva substitutes, or sialagogues) to eliminate their potential influence on symptom reporting.

Eligible individuals were invited to participate, informed of the study purpose and procedures, and provided written informed consent before enrollment.

### Measurements

Data were collected through a structured questionnaire, comprising sociodemographic variables, treatment-related factors, oral hygiene practices, oral clinical parameters, and oral hygiene status.

A single, skilled oral medicine specialist (F.E.) performed all clinical examinations and data collection processes; to guarantee uniformity, the specialist was calibrated in the use of the evaluation instruments. A previous study using complex oral lesion analysis supports the examiner’s diagnostic competence [27, 28]. A senior oncology consultant (H.W.) supervised the examiner to confirm clinical relevance and guarantee alignment with the patient’s oncologic history. This strategy was used to improve diagnostic reliability and reduce inter-examiner variability.

#### Sociodemographic and clinical variables

Information regarding age, gender, marital status, education level, employment status, household financial status (categorized as low, middle, or high based on self-reported monthly income), clinical history (tumor site, TNM stage, chemotherapy, and total radiation dose), elapsed time since completion of treatment (< 6 months vs. ≥6 months), and smoking status was collected through structured interviews and medical records review. All included participants had completed intensity-modulated radiotherapy (IMRT) as part of their primary oncologic treatment, which represents the standard radiotherapy protocol at our center during the study period.

#### Oral hygiene behavior and clinical oral health assessment

Oral hygiene practices (frequency of tooth-brushing, use of fluoride toothpaste, regular mouthwash usage, and receipt of professional oral hygiene instructions) were self-reported via structured interviews. Clinical oral hygiene status was assessed using the Oral Hygiene Index–Simplified (OHI-S), categorizing patients as having good (0–1.2), fair (1.3–3.0), or poor (3.1–6.0) oral hygiene. Clinical oral dryness was assessed using the Clinical Oral Dryness Score (CODS), a 10-item observational scale (range 0–10), where higher scores indicate more severe hyposalivation [29], and evaluations were performed by a calibrated dentist using standardized examination criteria.

#### Oral-health–related quality of life (OHRQoL)

OHRQoL was assessed using the validated Arabic version of the European Organization for Research and Treatment of Cancer Quality of Life Questionnaire–Head and Neck 35 (EORTC QLQ-H&N35) (Ouattassi et al., 2016). This questionnaire comprises 35 items covering pain, swallowing, speech, senses (taste/smell), social eating, social contact, sexuality, teeth, mouth opening (trismus), dry mouth, sticky saliva, coughing, and feeling ill, in addition to single-item measures of painkiller use, nutritional supplement intake, feeding tube dependency, and weight change. Scores were linearly transformed to a 0–100 scale, where higher scores indicated greater symptom severity and worse OHRQoL.

### Statistical analysis

Descriptive statistics (means, standard deviations, medians, interquartile ranges) summarized EORTC QLQ-H&N35 symptom scores. Data normality was evaluated using Shapiro-Wilk tests and Q–Q plots, showing moderate skewness; therefore, both parametric and non-parametric summaries were reported. A general linear model multivariate analysis of variance (GLM-MANOVA) was conducted in Stata (version 17.0, StataCorp LLC) to assess relationships between symptom scales and predictor variables. Multivariate normality and equality of variance–covariance matrices assumptions were verified. Predictors were initially evaluated in univariate (one-factor) models, followed by a comprehensive multifactorial model that simultaneously included all variables. One-way ANOVA or Kruskal–Wallis tests compared symptom scores across financial status and tumor sites, with homogeneity of variance tested using Levene’s method. No formal correction for multiple comparisons was applied, as the analyses were exploratory and intended to identify potential patterns for future hypothesis-driven study. Statistical significance was defined as *p* < 0.05. Missing data were handled using complete-case (listwise) analysis, whereby participants with missing values were excluded only from analyses involving the affected variable, and no imputation was performed.

## Results

**Fig. 1:**
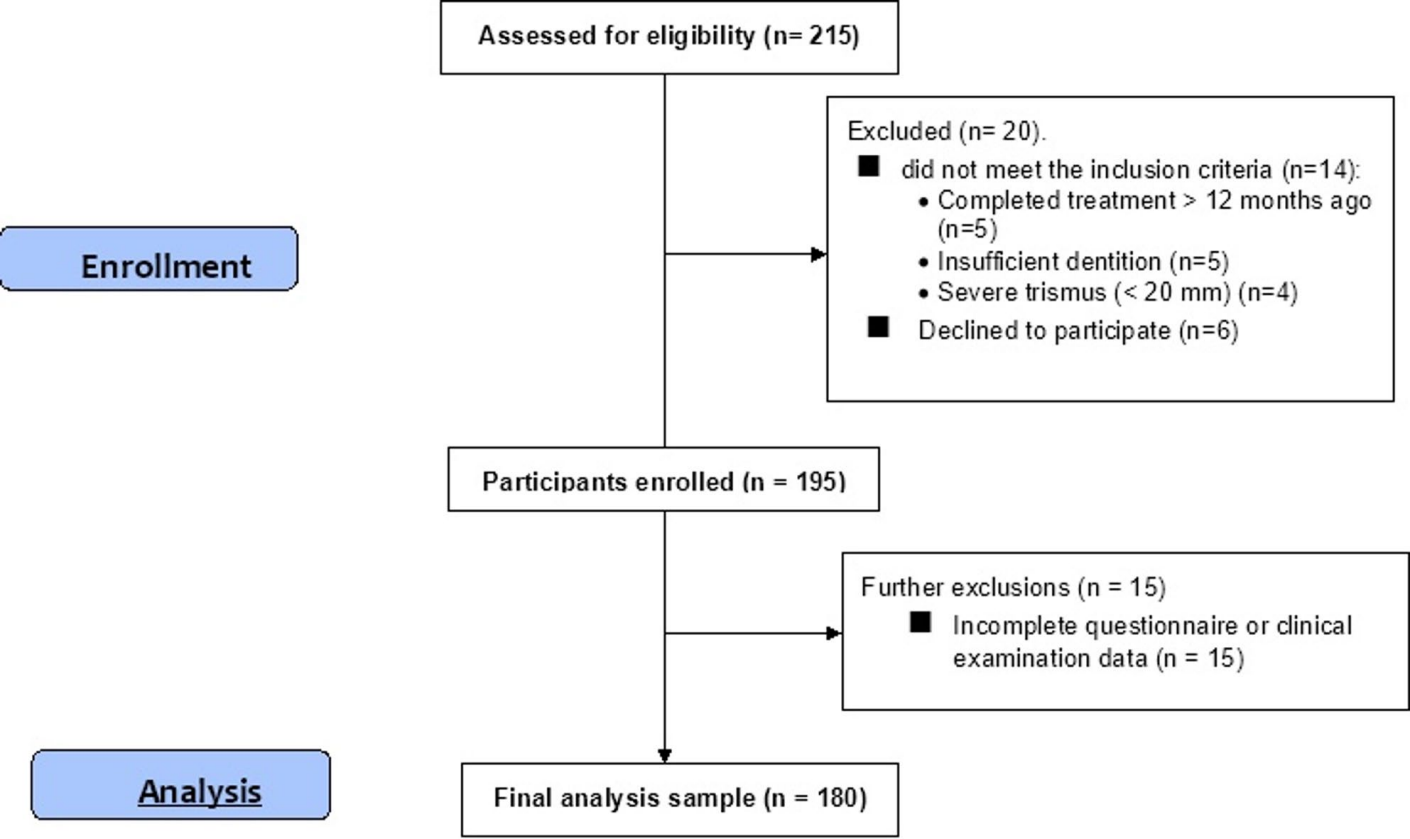
Participant flowchart following STROBE guidelines

A total of 215 survivors were screened; 20 were excluded due to ineligibility or refusal, and 195 were enrolled. After complete-case handling, the final analytic sample was 180 survivors **(**Fig. 1**).** Consecutive recruitment was extended through the planned window, resulting in a larger final sample than initially targeted, to ensure narrower confidence intervals and maintain statistical power for subgroup analyses.

### Descriptive data

#### Sociodemographic and clinical characteristics

Participants were predominantly middle-aged, male, and married, with most reporting middle-income status **(**Table 1**).** The oral cavity was the most frequent tumor site, and the majority had locally advanced disease. Nearly all survivors received IMRT, and more than half underwent concurrent chemoradiotherapy. Oral hygiene practices varied, and approximately half demonstrated fair-to-poor clinical oral hygiene.

**Table 1:** Sociodemographic, treatment related, habits, oral hygiene related factors of radiotherapy in HNC patients within 1 year, post cancer treatment

Variable	Category	*n* (%)
Age	< 52 years	86 (47.5%)
	≥ 52 years	94 (52.5%)
Gender	Male	135 (75.0%)
	Female	45 (25.0%)
Marital status	With spouse	146 (81.3%)
	Without spouse	34 (18.8%)
BMI	Under-weight	14 (7.5%)
	Normal weight	116 (65.0%)
	Over-weight	50 (27.5%)
Education level	Low	45 (25.0%)
	Middle	90 (50.0%)
	High	45 (25.0%)
Employment status	Employed	108 (60.0%)
	Unemployed	72 (40.0%)
Financial status	Low (≤ EGP 6,000 single/8,000 family)	41 (22.5%)
	Middle (EGP 6,001–13,000 single/8,001–18,000 family)	112 (62.5%)
	High (> EGP 13,000 single/18,000 family)	27 (15.0%)
Tumour site	Oral cavity	90 (50.0%)
	Oropharynx	45 (25.0%)
	Nasopharynx	23 (12.5%)
	Larynx	22 (12.5%)
Tumour stage at diagnosis	Stage I	11 (6.3%)
	Stage II	34 (18.8%)
	Stage III	56 (31.3%)
	Stage IV	79 (43.8%)
Chemotherapy	Yes	108 (60.0%)
	No	72 (40.0%)
Total radiation dose	≥ 70 Gy	130 (72.5%)
	< 70 Gy	50 (27.5%)
Elapsed time since treatment	< 6 months	81 (45.0%)
	≥ 6 months	99 (55.0%)
Smoking status	Current	63 (35.0%)
	Former	27 (15.0%)
	Never	90 (50.0%)
Brushing frequency	Twice daily	56 (31.3%)
	Once daily	79 (43.8%)
	Seldom	34 (18.8%)
	Never	11 (6.3%)
Mouthwash use	Yes	68 (37.5%)
	No	112 (62.5%)
Fluoride toothpaste use	Yes	124 (68.7%)
	No	56 (31.3%)
Oral-hygiene instructions given	Yes	108 (60.0%)
	No	72 (40.0%)
Oral Hygiene Index – Simplified (OHI-S)	Good	67 (37.5%)
	Fair	79 (43.8%)
	Poor	34 (18.8%)

#### EORTC QLQ-H&N35 symptom scores

**Fig. 2:**
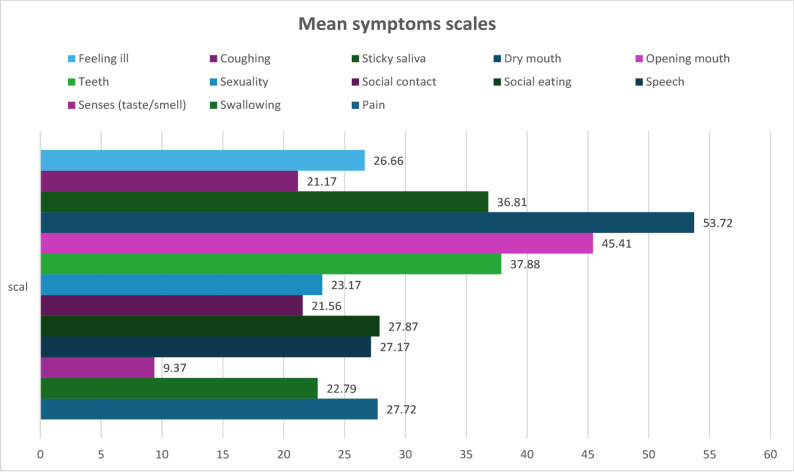
Mean scores of symptom scales assessed by the EORTC QLQ-H&N35 questionnaire in head-and-neck cancer survivors within one year post-treatment

Xerostomia-related symptoms (dry mouth and sticky saliva) represented the most prominent burden, followed by mouth-opening limitations and dental-related discomfort. Symptoms affecting swallowing, speech, and social eating were moderate, whereas sensory disturbance was the least severe. This pattern is illustrated in Fig. 2 and summarized in Table 2.

**Table 2:** Calculated scores of EORTC H&N35 scales for head and neck cancer survivors

Scale	Mean ± SD	Median	Range (min – max)
Pain	27.72 ± 11.67	28.46	1.00–100.00
Swallowing	22.79 ± 7.39	22.50	1.00–100.00
Senses (taste/smell)	9.37 ± 5.85	8.98	1.00–100.00
Speech	27.17 ± 15.49	26.97	1.00–100.00
Social eating	27.87 ± 16.88	27.08	1.00–100.00
Social contact	21.56 ± 14.74	18.69	1.00–100.00
Sexuality	23.17 ± 13.87	22.57	1.00–100.00
Teeth	37.88 ± 18.61	36.54	1.00–100.00
Opening mouth	45.41 ± 22.17	46.79	1.00–100.00
Dry mouth	53.72 ± 16.60	55.84	1.00–100.00
Sticky saliva	36.81 ± 20.08	35.27	1.00–100.00
Coughing	21.17 ± 13.20	21.00	1.00–100.00
Feeling ill	26.66 ± 11.57	26.03	1.00–100.00

### Main results

#### Multivariate determinants of symptom profile

In unadjusted models, several sociodemographic, clinical, and oral-health variables were associated with symptom patterns. In the multifactor model, financial status and tumor site remained independently associated with overall symptom burden, while brushing frequency demonstrated a marginal association (Table 3). These findings indicate that socioeconomic context and tumor location are key contributors to persistent post-treatment symptom severity.

**Table 3:** GLM-MANOVA test of the overall effect of the sociodemographic and clinical variables on the EORTC H&N35 scales

Variable	One-factor model (coefficient ± *p*-value)	Multifactor model (coefficient ± *p*-value)
Age	0.94 (*p* = 0.08)	0.96 (*p* = 0.21)
Gender	0.98 (*p* = 0.45)	0.99 (*p* = 0.68)
Marital status	0.95 (*p* = 0.12)	0.96 (*p* = 0.25)
BMI	0.91 (*p* = 0.03*)	0.94 (*p* = 0.09)
Education level	0.89 (*p* = 0.02*)	0.91 (*p* = 0.06)
Employment status	0.96 (*p* = 0.17)	0.97 (*p* = 0.31)
Financial status	0.87 (*p* = 0.01*)	0.90 (*p* = 0.04*)
Tumour site	0.83 (*p* = 0.004*)	0.88 (*p* = 0.02*)
Tumour stage	0.94 (*p* = 0.09)	0.96 (*p* = 0.18)
Chemotherapy	0.97 (*p* = 0.26)	0.98 (*p* = 0.38)
Total radiation dose	0.99 (*p* = 0.74)	0.99 (*p* = 0.82)
Elapsed time since treatment	0.92 (*p* = 0.05)	0.95 (*p* = 0.12)
Smoking	0.91 (*p* = 0.03*)	0.93 (*p* = 0.08)
Brushing frequency	0.88 (*p* = 0.01*)	0.91 (*p* = 0.05)
Mouthwash use	0.97 (*p* = 0.20)	0.98 (*p* = 0.33)
Fluoride-toothpaste use	0.98 (*p* = 0.37)	0.99 (*p* = 0.57)
Instructions by hygienist	0.95 (*p* = 0.11)	0.96 (*p* = 0.24)
Oral Hygiene Index (OHI-S)	0.89 (*p* = 0.02*)	0.91 (*p* = 0.06)
CODs†	0.92 (*p* = 0.04)	0.94 (*p* = 0.10)

*GLM-MANOVA* General linear model multivariate of variance

The one factor model: only one independent variable was entered into the model

The multifactor model: all mentioned variables were entered as independent variables in the model

### Other analyses

#### Financial status comparisons

**Fig. 3:**
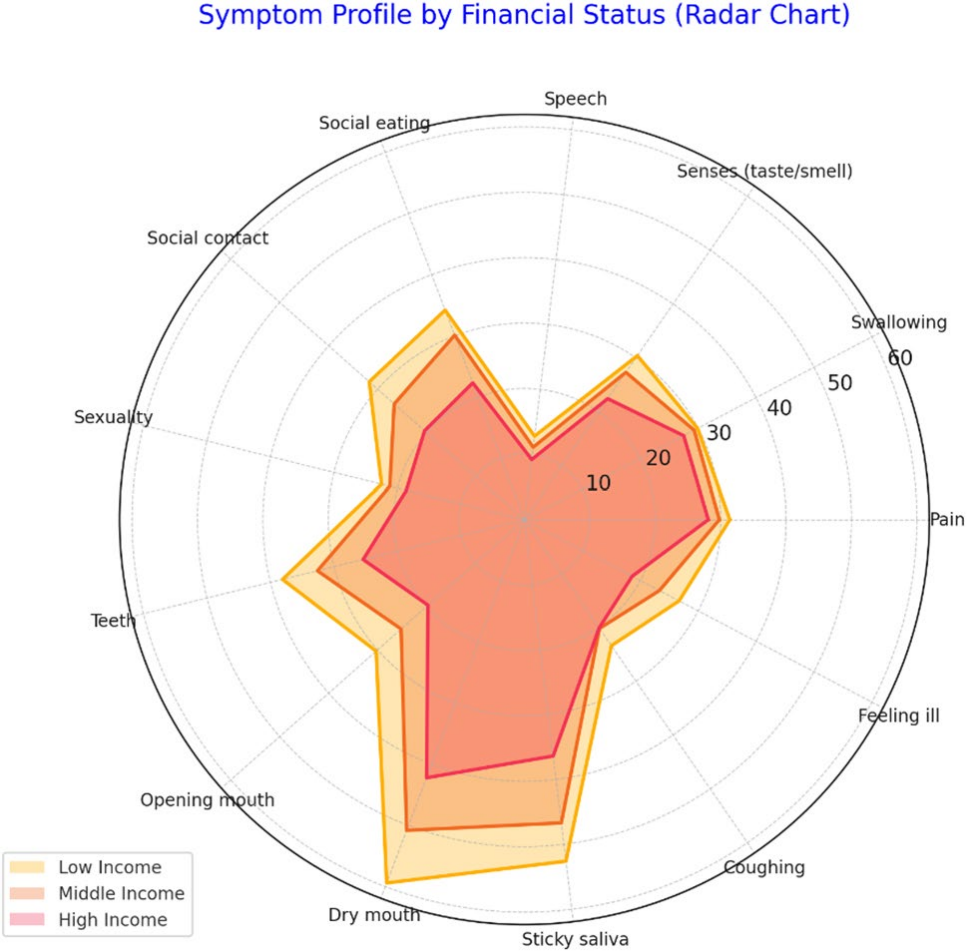
Radar chart comparing symptom profiles across financial status groups (low, middle, and high income) using the EORTC QLQ-H&N35

Survivors with lower financial status consistently experienced greater symptom burden, particularly in xerostomia, sticky saliva, oral function, and social functioning domains. The magnitude and precision of group differences are illustrated by the 95% confidence intervals reported in Table 4. This gradient is visually represented in Fig. 3, reflecting a compounded impact of economic disadvantage on recovery and rehabilitation.

**Table 4:** The comparisons of EORTC H&N35 scales for head and neck cancer survivors at different financial status levels

Scale	Low (*n* = 18) Mean ± SD (95% CI)	Middle (*n* = 50) Mean ± SD (95% CI)	High (*n* = 12) Mean ± SD (95% CI)	*p*-value
Pain	31.4 ± 8.1 (27.7–35.1)	29.8 ± 6.5 (28.0–31.6)	28.1 ± 5.2 (25.2–31.0)	0.048*
Swallowing	29.9 ± 7.5 (26.4–33.4)	29.3 ± 6.9 (27.4–31.2)	27.5 ± 5.7 (24.3–30.7)	0.217
Senses (taste/smell)	30.4 ± 8.0 (26.7–34.1)	27.3 ± 6.8 (25.4–29.2)	22.4 ± 4.9 (19.6–25.2)	0.006*
Speech	12.8 ± 4.3 (10.8–14.8)	11.1 ± 3.9 (10.0–12.2)	9.2 ± 3.0 (7.5–10.9)	0.012*
Social eating	34.2 ± 9.7 (29.7–38.7)	30.1 ± 8.8 (27.7–32.5)	22.3 ± 6.9 (18.4–26.2)	< 0.001*
Social contact	31.7 ± 10.2 (27.0–36.4)	26.6 ± 8.7 (24.2–29.0)	20.4 ± 7.5 (16.2–24.6)	< 0.001*
Sexuality	22.5 ± 9.1 (18.3–26.7)	21.2 ± 8.0 (19.0–23.4)	18.6 ± 6.1 (15.1–22.1)	0.082
Teeth	38.1 ± 8.7 (34.1–42.1)	32.6 ± 7.9 (30.4–34.8)	25.4 ± 6.8 (21.6–29.2)	< 0.001*
Opening mouth	30.3 ± 7.5 (26.8–33.8)	25.2 ± 6.2 (23.5–26.9)	19.7 ± 5.1 (16.8–22.6)	< 0.001*
Dry mouth	59.4 ± 10.5 (54.5–64.3)	50.8 ± 9.7 (48.1–53.5)	42.2 ± 8.2 (37.6–46.8)	< 0.001*
Sticky saliva	52.6 ± 11.2 (47.4–57.8)	46.7 ± 10.3 (43.8–49.6)	36.4 ± 9.0 (31.3–41.5)	< 0.001*
Coughing	23.4 ± 6.9 (20.2–26.6)	20.2 ± 6.4 (18.4–22.0)	20.1 ± 5.7 (16.9–23.3)	0.139
Feeling ill	26.7 ± 7.5 (23.2–30.2)	23.3 ± 6.1 (21.6–25.0)	18.6 ± 5.2 (15.7–21.5)	0.005*

Bold p-values denote statistical significance at α = 0.05

†Kruskal–Wallis test across the four tumour-site groups

#### Tumor-site comparisons

**Fig. 4:**
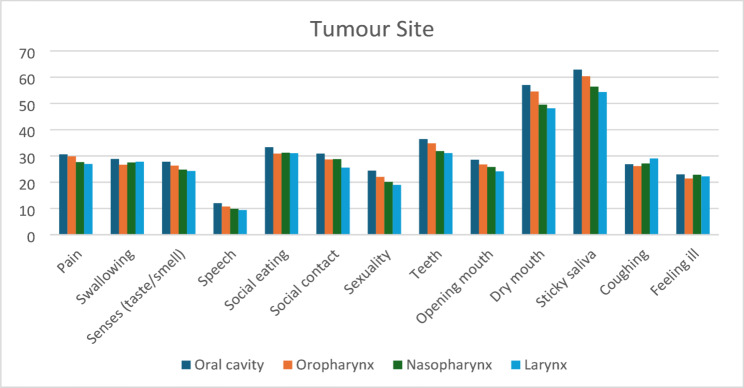
Comparison of mean symptom scores across different tumour sites (oral cavity, oropharynx, nasopharynx, larynx) measured by the EORTC QLQ-H&N35 questionnaire

Survivors with oral cavity cancers reported the highest levels of functional and xerostomia-related impairment, whereas those with laryngeal cancers generally reported the lowest burden **(**Table 5**).** These differences, illustrated in Fig. 4, likely reflect anatomical site involvement, radiotherapy field distribution, and associated effects on salivary gland and oral functional structures.

**Table 5:** The comparisons of EORTC H&N35 scales for head and neck cancer survivors at different tumor sites

Scale	Oral cavity (*n* = 40) Mean ± SD (95% CI)	Oropharynx (*n* = 20) Mean ± SD (95% CI)	Nasopharynx (*n* = 10) Mean ± SD (95% CI)	Larynx (*n* = 10) Mean ± SD (95% CI)	*p*-value†
Pain	30.62 ± 6.12 (28.7–32.5)	29.86 ± 6.53 (27.0–32.7)	27.67 ± 5.71 (24.1–31.2)	26.94 ± 5.65 (23.4–30.4)	0.046*
Swallowing	28.82 ± 6.13 (26.9–30.7)	26.64 ± 6.74 (23.7–29.6)	27.49 ± 5.44 (24.1–30.9)	27.80 ± 5.71 (24.3–31.3)	0.534
Senses (taste/smell)	27.78 ± 6.14 (25.9–29.7)	26.32 ± 6.93 (23.3–29.4)	24.79 ± 5.95 (21.1–28.5)	24.25 ± 5.49 (20.8–27.7)	0.218
Speech	11.99 ± 3.56 (10.9–13.1)	10.72 ± 3.81 (9.1–12.4)	9.85 ± 3.25 (7.8–11.9)	9.40 ± 2.81 (7.7–11.1)	0.041*
Social eating	33.33 ± 7.01 (31.2–35.5)	30.90 ± 7.25 (27.7–34.1)	31.22 ± 6.94 (26.9–35.5)	31.04 ± 6.57 (27.0–35.1)	0.651
Social contact	30.87 ± 7.09 (28.7–33.1)	28.64 ± 7.38 (25.4–31.9)	28.81 ± 7.08 (24.4–33.2)	25.57 ± 6.61 (21.5–29.7)	0.048*
Sexuality	24.42 ± 6.38 (22.4–26.4)	22.03 ± 6.29 (19.3–24.8)	20.13 ± 6.19 (16.3–24.0)	18.97 ± 6.08 (15.2–22.7)	0.024*
Teeth	36.44 ± 6.17 (34.5–38.4)	34.79 ± 6.71 (31.8–37.7)	31.86 ± 6.49 (27.8–35.9)	31.06 ± 5.84 (27.4–34.7)	0.022*
Opening mouth	28.54 ± 5.42 (26.9–30.2)	26.76 ± 5.42 (24.4–29.1)	25.79 ± 5.10 (22.6–29.0)	24.13 ± 4.72 (21.2–27.1)	0.030*
Dry mouth	57.01 ± 9.87 (54.0–60.1)	54.53 ± 10.31 (50.0–59.0)	49.52 ± 9.36 (43.7–55.3)	48.13 ± 9.05 (42.5–53.7)	0.008*
Sticky saliva	62.90 ± 10.10 (59.8–66.0)	60.33 ± 10.62 (55.7–65.0)	56.41 ± 9.83 (50.3–62.5)	54.35 ± 9.26 (48.6–60.1)	0.035*
Coughing	26.84 ± 6.38 (24.9–28.8)	26.11 ± 6.73 (23.2–29.1)	27.13 ± 6.67 (23.0–31.3)	29.03 ± 5.91 (25.4–32.7)	0.385
Feeling ill	22.96 ± 6.03 (21.1–24.8)	21.39 ± 6.25 (18.7–24.1)	22.81 ± 5.84 (19.2–26.4)	22.21 ± 6.15 (18.4–26.0)	0.731

Bold p-values denote statistical significance at α = 0.05

†Kruskal–Wallis test across the four tumour-site groups

## Discussion

This is the first study in Egypt using the validated, comprehensive EORTC QLQ-H&N35 questionnaire to assess the OHRQoL among HNC survivors.

### Key findings

It was found that the most severe patient-reported problems on the EORTC QLQ-H&N35 were dry mouth, jaw function impairment, and dental problems. Specifically, mean scores for dry mouth (≈ 53.7), difficulty opening the mouth (≈ 45.4), and teeth problems (≈ 37.9) were substantially higher than for other symptoms, indicating these issues are particularly pronounced. In contrast, symptoms such as overall malaise, coughing, and loss of taste/smell had relatively low scores (mean ≈ 9–27). Thus, our primary finding is that oral dryness and functional limitations dominate the oral health-related quality-of-life profile in early survivorship.

Multivariate analysis further showed that tumor site, socioeconomic status, and oral hygiene habit**s** were the strongest predictors of symptom burden. In adjusted models, lower financial status, oral-cavity primary tumors were significantly associated, and infrequent tooth-brushing was marginally significantly associated with higher (worse) symptom scores. Notably, survivors with oral-cavity cancers reported significantly greater impairments in speech, swallowing, and salivary functions than those with laryngeal or nasopharyngeal tumors.

In summary, our survivors’ worst complaints - dry mouth, trismus, and dental pain - are exacerbated by oral-cavity disease and by low income and poor oral hygiene practices. These results indicate a significant gap in oral care following treatment and suggest modifiable risk factors that may guide focused supportive interventions in settings with limited resources, like Egypt.

### Comparison with literature

These findings align with global literature on HNC survivors. In a systematic review of 37 studies on 1-year post-treatment survivors, it was noted that fatigue, dry mouth, and sticky saliva remain as outstanding problems [30]. For instance, the OraRad multicenter study (*n* ≈ 572) reported that dry mouth and sticky saliva were the most persistently elevated quality-of-life variables up to 24 months post-RT, especially worsening during the first 6–12 months [31]. Similarly, [32] found that RT techniques were significantly related to H&N35 symptoms, including teeth problems, mouth opening, dry mouth, and sticky saliva [32], which underlines their direct link to treatment.

Our observation that oral cancer leads to worse outcomes was in line with [33], who reported significant tumor-site differences on 10 of 13 H&N35 scales, indicating site-specific symptom patterns [33]. We similarly found that oral-cavity primaries drove deficits in speech and swallowing domains. Beyond QLQ-H&N35 findings, other tools corroborate reduced oral QOL in HNC survivors. For example, [34] compared oral cancer patients ≥ 6 months post-treatment to healthy controls using the OHIP-14 and found significantly worse oral health-related QOL (poorer OHIP-14 scores) in the patient group [34]. Likewise, studies using the FACT-H&N instrument demonstrated that factors such as low income, comorbidities, and physical symptoms accounted for the majority of variance in QoL scores [35], echoing our findings on the impact of socioeconomic and health variables.

### Comparison with studies from Low- and Middle-Income countries

Our findings align with a growing body of evidence from other LMICs on common post-treatment problems shaped by resource constraints. The symptom triad of xerostomia, trismus, and dental problems, which dominates our cohort’s experience, is a recurrent theme in recent literature. Most recently, the longitudinal Brazilian study by Caminha et al. (2024) demonstrated a significant worsening of functional limitation and physical pain during radiotherapy that persisted after its completion, thus clearly delineating the treatment-induced deterioration of OHRQoL [36]. In this respect, a recently published cross-sectional study from Pakistan by Qamar et al. (2024) [24], also using the EORTC QLQ-H&N35, identified almost identical primary burdens to our own, with survivors within the first year post-treatment reporting dry mouth (67.1), sticky saliva (52.3), and teeth problems (45.2) as the most severe issues. This remarkable congruence underlines the universality of salivary and functional sequelae.

Additionally, a Nigerian study by Nduagu et al. (2022) highlighted some of the systemic issues: that late-stage diagnosis and inadequate pre-radiation dental care significantly exacerbate posttreatment complications in survivors and directly impair their quality of life [37].

These observations were further cemented through studies from different LMIC regions in the early part of the last decade. A case-control study among Brazilians by Stuani et al. (2018) previously established that the functional limitation and physical pain dimensions of the OHIP-14 were significantly worse among post-treatment HNC patients than among pre-treatment patients and cancer-free controls [38].

Looking further back, research from India has repeatedly underlined the contribution of socioeconomic factors. Shavi et al. (2015) highlighted the low-income status and low educational attainment as strong predictors of poor OHRQoL, since such patients were those who could not afford rehabilitative care [11]. This supports earlier findings by Chaukar et al. (2009), who found that close to 40% of HNC survivors in India reported financial burden associated with treatment costs, which had a direct correlation with poorer quality of life outcomes [39].

Survivors of HNC in resource-poor environments universally experience a similar cluster of extremely debilitating oral symptoms. These are strongly influenced by poverty, the lack of access to preventative dentistry, and supportive care pathways that are poorly developed.

### Clinical implications

Clinically, these results highlight key targets for intervention in HNC survivors. The prominence of dry mouth and trismus suggests that proactive measures to preserve salivary function and jaw mobility are essential. Previous studies recommend regular monitoring for dry mouth and related symptoms, and the development of effective interventions to address them [40–45].

In practice, this might include saliva-stimulant medications, saliva substitutes, meticulous oral hygiene and hydration, and jaw-stretching exercises or devices. Our finding that low income predicts a worse symptom burden is also important: it implies that socially disadvantaged survivors may need extra support (e.g., nutritional assistance, subsidized dental care) to prevent exacerbation of oral health problems. Indeed, [46] similarly reported that patients with lower annual income had significantly lower global QOL scores [46]. From a technical standpoint, our data reinforce the benefits of modern RT approaches: as [32]. showed, IMRT markedly improves outcomes on HN35 scales, including dry mouth and opening mouth [32]. These considerations underscore the need for a multidisciplinary survivorship model care integrating dental oncology, speech/swallow therapy, nutrition, and social support to mitigate the long-term oral health consequences of head-and-neck cancer treatment.

The strengths of our study include the use of a validated, cancer-specific QOL instrument (EORTC QLQ-H&N35) and the integration of clinical oral health measures (OHI-S hygiene index and clinical oral dryness score) to provide objective correlates of patient-reported symptoms. Our comprehensive multivariate analysis also adjusted for a broad range of sociodemographic and clinical factors.

Another important finding is that the use of objective clinical measures, such as OHI-S and CODS, was significant in univariate analyses but lost their significance in the final adjusted model. This likely indicates that the influence of these factors on patient-reported OHRQoL is not direct but largely mediated or explained by other factors. For example:

#### Mediation by behavior

The patient’s frequency of tooth-brushing (which remained a significant predictor) directly determines their OHI-S score. The behavior, in the multivariate context, appears to be a stronger predictor of symptom experience than the resulting clinical state (plaque and calculus), perhaps because it captures motivation, self-efficacy, and overall engagement in self-care, which broadly influence health perceptions.

#### Confounding by socioeconomics

 Economic status, which is a strong independent predictor, affects oral hygiene and clinical dryness. Low income might limit access to fluoride toothpaste, soft toothbrushes, professional dentistry, and hydration aids, all of which affect OHI-S and CODS negatively. Once economic status is controlled, the unique contribution of clinical signs becomes smaller, as they share a common cause.

#### Subjective versus objective experience

 This finding illustrates a critical trait of quality of life-it is inherently subjective. Whereas CODS is an objective clinical measure of dryness, patient bother from xerostomia is filtered through coping mechanisms, expectations, and overall symptom burden. The model suggests that the financial context and hygiene practices of a patient are superior proxies of this subjective burden to the clinical sign in isolation.

##### In summary

the worst complaints of our survivors are most directly shaped by the site of their cancer, their socioeconomic resources, and their daily health behaviors. The loss of significance of OHI-S and CODS after adjustment does not mean oral health is unimportant; rather, it reveals that the pathway to poor OHRQoL runs through modifiable socioeconomic and behavioral channels, with clinical signs being intermediate outcomes on that pathway.

#### Limitations

The study has several limitations that should be considered when interpreting the results. First, the current study’s cross-sectional and single-center design restricts the ability to conclude causality and limits its generalizability. Second, the distribution of tumor sites in our cohort (with the majority being cancers of the oral cavity) may reflect specific referral patterns of our tertiary care center rather than the true national epidemiology of HNC in Egypt, which may impact the generalizability of our results. Third, the lack of pre-treatment baseline QOL data limits our ability to measure the direct effects of treatment and assess how individuals change over time. Fourth, despite evaluating a large number of predictors, we did not formally correct for multiple comparisons because the study was exploratory, and the sample size was modest (*n* = 180). Fifth, self-reported oral hygiene practices may be subject to recall bias. Finally, we evaluated results only during the first year after treatment; longer-term monitoring is required to determine whether these symptoms persist or evolve.

#### Future research

 Prospective longitudinal studies with pre-treatment baselines are needed to map trajectories of OHRQoL. Interventional trials are required to target the main troublesome domains, such as new radioprotectors to prevent xerostomia, or intensive rehabilitative exercises to reduce trismus and dysphagia. It is necessary to assess how comprehensive oral care programs, including dental rehabilitation and hygiene support, can enhance quality of life. Studies should investigate how supportive services (financial counseling, nutrition programs, and psychosocial support) can mitigate the effects of treatment in light of the socioeconomic disparities. Simultaneously, more psychometric research could improve OHRQoL measurement in HNC. Lastly, studies on how comorbidities, lifestyle factors, and cancer treatment interact will help customize follow-up care to each patient’s unique risk profile.

## Conclusion

The current study reveals an important burden of oral health-related quality-of-life impairments among head and neck cancer survivors, with xerostomia, trismus, and dental complications emerging as the most significant obstacles that were reported in the first year after radiation therapy. The multivariate analysis found that tumor site, socioeconomic status, and oral hygiene practices were the most significant independent predictors of symptom severity. These results highlight the critical need for integrated, multidisciplinary survivorship care models that incorporate proactive dental support, especially for patients with oral cancer and from socioeconomically deprived backgrounds. Prioritizing resource-sensitive supportive services and customized oral health interventions will enhance long-term results and equity in cancer recovery. Addressing modifiable risk factors through integrated oral care and policy reform is essential to improve survivorship outcomes in low-resource settings.

## Data Availability

The datasets generated and analyzed during the current study are available from the corresponding author upon reasonable request.
